# Impacts of Climate Change on Native Landcover: Seeking Future Climatic Refuges

**DOI:** 10.1371/journal.pone.0162500

**Published:** 2016-09-12

**Authors:** Marina Zanin, Ana Luisa Mangabeira Albernaz

**Affiliations:** Earth Sciences and Ecology Center, Emílio Goeldi Museum of Pará, Av. Perimetral, 1901—Terra Firme, CEP 66077–830, Belém, PA, Brasil; University of Vigo, SPAIN

## Abstract

Climate change is a driver for diverse impacts on global biodiversity. We investigated its impacts on native landcover distribution in South America, seeking to predict its effect as a new force driving habitat loss and population isolation. Moreover, we mapped potential future climatic refuges, which are likely to be key areas for biodiversity conservation under climate change scenarios. Climatically similar native landcovers were aggregated using a decision tree, generating a reclassified landcover map, from which 25% of the map’s coverage was randomly selected to fuel distribution models. We selected the best geographical distribution models among twelve techniques, validating the predicted distribution for current climate with the landcover map and used the best technique to predict the future distribution. All landcover categories showed changes in area and displacement of the latitudinal/longitudinal centroid. Closed vegetation was the only landcover type predicted to expand its distributional range. The range contractions predicted for other categories were intense, even suggesting extirpation of the sparse vegetation category. The landcover refuges under future climate change represent a small proportion of the South American area and they are disproportionately represented and unevenly distributed, predominantly occupying five of 26 South American countries. The predicted changes, regardless of their direction and intensity, can put biodiversity at risk because they are expected to occur in the near future in terms of the temporal scales of ecological and evolutionary processes. Recognition of the threat of climate change allows more efficient conservation actions.

## Introduction

Native landcover alterations have reached alarming rates in the last four decades [1,2] and changes are expected to increase in the near future, exacerbating threats to biodiversity. However, most of our current attention is directed toward losses of native landcover due to agricultural or urban expansion, overlooking other causes of reduction. Human-induced climate change can be a new driver of losses in native landcover because native vegetation distributions are related to climatic variables and, consequently, they may be affected by future climate change [3–7]. So, while native vegetation conversion might be the main anthropogenic change currently threatening biodiversity, climate change could be equally or even more influential in the near future [8], exacerbating the negative consequences of native vegetation losses.

Landcover alteration mediated by future climate change could become a direct threat to biodiversity due to its potential to change habitat availability for species, reducing or displacing them [9].There are also indirect consequences because landcover categories correspond to different habitat structures and, when unfavorable, these latter can act as barriers to species movement [9]. In this way, landcover changes through space and time can define boundaries of present and future ecological regions suitable for species [10]. Recognition of these boundaries can promote new perspectives on management of anthropogenic climate change impacts [11,12], since we can identify future refuges in which species can persist. Consequently, understanding the loss or displacement of landcover types is a high priority for research and for supporting conservation actions [13,14].

The conceptual and methodological framework to study effects of climate change on biodiversity has been advancing in recent years, driven by the importance and complexity of the topic [14–18]. These approaches involve a diversity of subjects, which include forest dynamics and threatened and specialist species. One of the main approaches employed to study climate change impacts has typically consisted of a technique known as species distribution modeling. It is also known as ecological niche modeling because it aims to predict the potential distribution of species through their fundamental niches [19]. As a result, species distribution patterns, mediated by climate change, are one of the most studied themes in climate change science [20–22].

However, the species distribution models can be used to target predictions beyond species ecological niches because, in a simplified view, these methods consist of correlatives approaches between occurrence and environmental data (frequently climatic variables). In this way, species distribution models identify a multi-dimensional environmental space, which is projected in a two or three-dimensional map, showing the expected geographic distribution based on the environmental conditions [19]. Therefore, the possibilities of using species distribution models concern the investigation of environmental mediated patterns beyond the ecological attributes of species, which permitted the use of these techniques even in other research areas (in social science [23], reconstructing Late Quaternary vegetation [7], mapping the malaria risk in Africa [24], investigating the distribution of archeological sites [25]). Because we adopted a non-species-specific approach in our study, we term our approach geographic distribution modelling.

The use of native landcover as a focus of analysis brings some methodological advantages, which increase analytical robustness. Unlike species distributions, landcover distributions are well known, which improves at least two methodological steps of geographical distribution modeling. One of the main problems of studies using geographical distribution modeling is the sampling bias associated with species occurrences (the basic information used to estimate a species’ fundamental niche), which lead to biased predictions of distribution [26,27]. The second is that validation data usually consist of a subset of the entire dataset, so that the validation of predicted distributions is also biased (an essential step of geographical distribution modeling) [28,29]. As an alternative approach, the known landcover distribution enables part of the data to be used to generate the prediction (calibration) and the remaining data can be used to validate the distribution essentially bias-free. These methodological advantages confer benefits in terms of applicability to conservation because planning for the future must be based on the most reliable results, which infers predictions with reduced uncertainty and distribution errors. Therefore, investigation of native landcover changes in future climate scenarios has crucial applicability for public policy on biodiversity conservation because of the methodological robustness with which predictions can be generated.

Therefore, we study the effects of future climate scenarios on native landcover distribution in South America using the geographical distribution modeling approach and infer the conservation implications of our findings. The study area includes at least seven major terrestrial biomes depending on the classification adopted (tropical forest, deciduous forest, shrubland, grassland, savanna, desert, and mountain), which are distributed in 13 countries (Argentina, Bolivia, Brazil, Chile, Colombia, Ecuador, French Guiana, Guyana, Paraguay, Peru, Suriname, Uruguay, and Venezuela). Even though ecological patterns do not respect sociopolitical boundaries, many conservation strategies are undertaken at this scale, highlighting the importance of analyzing and discussing the effects of climate change at the country level. We also endeavor to identify the climatic refuges of native landcover that could be essential for protecting biodiversity into the future and discuss the relative importance of them in each of the South American countries. To do this, we asked the following questions: what are the native landcover categories that will potentially contract or expand their distributions as a consequence of climate change, resulting in habitat loss or habitat expansion?; what is the direction and intensity of these potential changes (latitudinal/longitudinal), leading to potential raising of barriers to species dispersal?; and which regions and countries are the most stable, indicating that they should be prioritized for conservation?

## Materials and Methods

### Database

South America’s landcover was categorized according to GlobCover (available at http://due.esrin.esa.int/page_globcover.php), one of the most widely used in ecological studies [30] due to its ability to correctly describe current landcover and for its applicability to large-scale studies. The GlobCover map is a categorization of MERIS FR mosaics for the year 2009 through an automatic and regionally-tuned classification, which resulted in 22 landcover classes, 19 of which occur in South America (S1 Fig). This landcover map follows the classes defined by the United Nations Land Cover Classification System.

In our study, anthropogenic landcover categories (urban areas, agricultural lands, etc.) were not included in any of the analyses because their distribution, current and into the future, are strongly influenced by political incentives for urban/agricultural development, roads, etc. As a result, anthropogenic landcover categories must be analyzed using variables that are beyond the scope of the climatic variables used in this paper. For this reason, we removed all categories under anthropogenic influence (urban areas, agricultural lands, etc.) from the landcover map before modeling.

The landcover map was overlaid with grid cells of 0.5° latitude and longitude. Then, the landcover map was upscaled according to the most abundant native landcover category in each grid cell. There is an error associated with this extrapolation because the current most abundant native landcover does not always represent the original vegetation, mainly in grid cells located in agricultural lands. However, this error did not affect our results, as is described below in the results section.

We investigated nineteen climatic variables to evaluate potential landcover distributions (S1 Table; data available from http://ecoclimate.org). We chose three different Global Climate Models (GCMs: Community Climate System Model—CCSM, Model for Interdisciplinary Research on Climate–MIROC, and Institut Pierre Simon Laplace—IPSL) to decrease uncertainty based on inconsistencies between climate models [31]. These GCMs were selected because they have widely available digital databases, predict climate change differently (which helps reveal to what extent results depend on the CGM chosen), and exhibit a reasonable adjustment over the study region. All three were derived from the Coupled Model Intercomparison Project–Phase 5 (CMPI5) [32].

We adopted the pre-industrial climatic scenario as being responsible for current native landcover distributions. Changes to native landcover distributions since the industrial revolution are mainly due to urban and agricultural expansion and not to climate change because there is a time lag in the response of landcover to climate change [33,34]. Therefore, we assert that current native landcover is better characterized by the pre-industrial climatic scenario, justifying our choice. Climate data up to the year 2080 was generated according to the emission scenarios of the Representative Concentration Pathway (RCP) 4.5 and 8.5.

### Landcover and climatic distribution similarity

**Fig 1:**
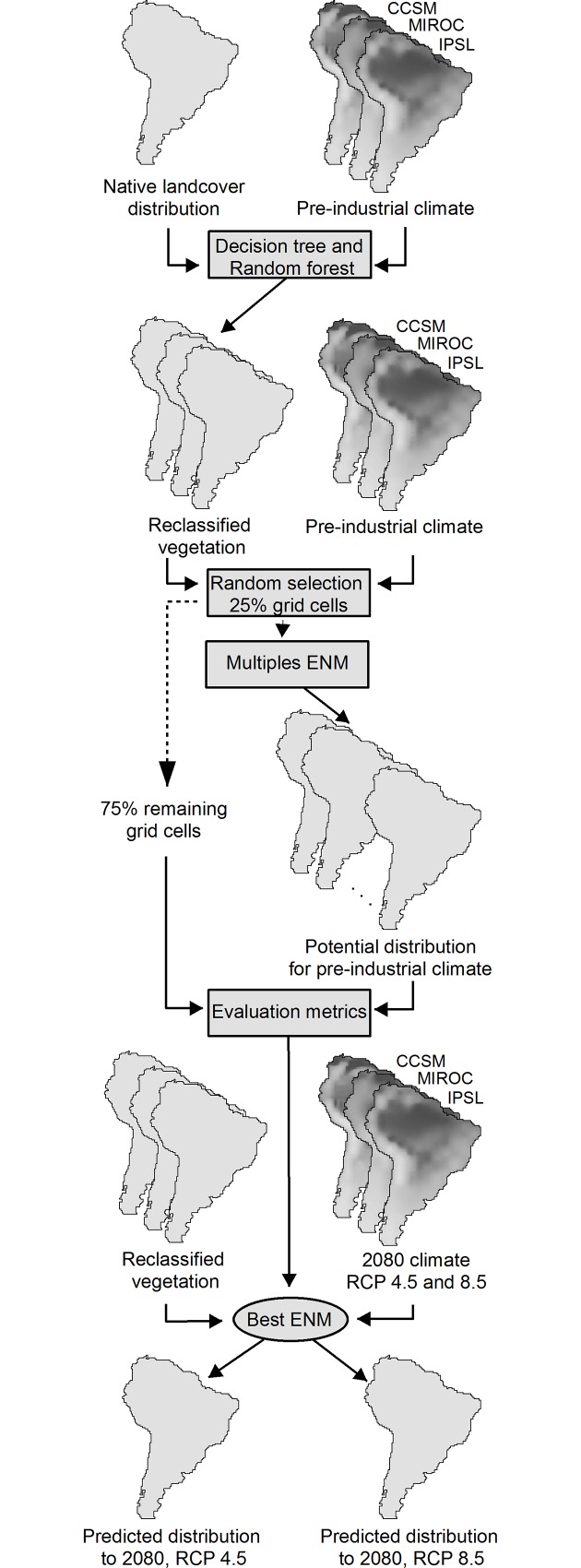
Steps of the methodological approach used in this study (GDM–geographical distribution model). Methods.

Since our approach includes only climatic variation, not considering the full range of variables influencing the distribution of landcovers, it is expected that some landcover classes show the same environmental/climatic space. This means that two or more landcover classes can be described by the same climatic conditions because other variables, such as topography and soils, can be limiting of their distribution. Therefore, we first evaluated climatic similarities among grid cells, creating groups that represent landcover categories that have a unique climatic identity (Fig 1). It allowed us to characterize new landcover classes according to climatic variables, thereby enabling our modeling approach.

The classical way to group data according to ecological similarity is by using cluster analysis [35]. Cluster analysis is not a specific algorithm, but a conceptual method that generates groups based on the similarity of sampling units. The most common clustering algorithms are k-means and Unweighted Pair Group Method with Arithmetic Mean (UPGMA), which are examples of non-hierarchical and hierarchical methods, respectively [35]. However, these methods can create arbitrary groups because they depend on *a priori* definitions of group numbers in the case of k-means or the cut-off point of a dendrogram for UPGMA [35].

Here, we used a decision tree algorithm to generate grid cell groupings based on climate similarity between native landcover categories. The decision tree is an approach only recently employed in ecological studies, frequently with other objectives such as sensitivity analysis [36,37] and classification of species according to extinction risk [38,39]. Decision trees have the conceptual logic of cluster analyses, which is the allocation of sampling units to a group possessing greater similarities, thereby increasing intra-group homogeneity and between-group heterogeneity [40].

The decision tree is a logical model, so named due to its graphical representation in binary tree form, which shows how the response variable (in this case, the native landcover category) can be predicted by explanatory variables (here, the climatic variables) [40]. The decision tree algorithm divides the initial dataset (grid cells) into homogeneous subsets, with one variable in each subdivision step (called in decision tree terminology a *node*), until homogeneous and indivisible subsets remain (*leaves*), which are our clusters [40]. Due to its progressive approach, the decision tree can be considered a hierarchical method, but starting with only one group (all sampling units) that is progressively subdivided to maximize the similarity within groups [40]. This is the opposite approach to UPGMA whereby each sampling unit is progressively aggregated until only one group remains.

The result of the decision tree process is a highly subdivided data set, which can partition grid cells with the same landcover type into more than one *leaf*. Each *leaf* has a prediction that best characterizes its landcover category, i.e. it may or may not be the original category of the grid cell. This prediction reveals which landcover categories have a distribution corresponding to the climate and for which categories the climatic space overlap, resulting in a shared classification. This is the basic evaluation about the ability of landcover distribution to be predicted by climate that is essential for our approach.

The decision tree was carried out singly for each climate model. To avoid over-fitting in the decision tree results, we evaluated the optimal number of subdivisions through 10,000 cross-validations, which measured the relative error with the addition of a new subdivision (*node*); in other words, a balance between accuracy of prediction and model complexity [40]. Due to cross-validation removing unnecessary subdivisions and the use of one variable for each step (a characteristic of the decision tree), there is no risk of overparameterization because the method selects only the most efficient variables for creating groups [40]. To evaluate the efficiency of the decision tree to correctly classify landcover categories, we conducted a random forest with 10,000 random trees generated by bootstrap sampling. The analyses were conducted in R software, using the *rpart* [41] and *randomForest* packages [42].

Finally, we evaluated if the efficiency of the classification was affected by expansion of anthropogenic lands. We performed the decision trees after removing grid cells with more than 50% of area occupied by anthropogenic landcover and compared the proportion of grid cells correctly classified with the result using all grid cells. We also related the probability of a grid cell to be correctly classified with the proportion of anthropogenic landcover in the cell.

### Modeling approach

To predict the native landscape cover distribution, we selected nine different species distribution methods and three ensemble forecastings. Each ensemble was composed by a different group of methods: (i) bioclimatic envelope and distance-based models–BIOCLIM [43], Gower distance [44], Mahalanobis distance [45]; (ii) statistical models—Generalized Linear Models (GLM) [46], Generalized Additive Models (GAM) [47], Multivariate Adaptive Regression Splines (MARS) [48]; and (iii) machine-learning models—Maximum Entropy (MaxEnt) [49], Genetic Algorithm for Rule Set Production (GARP) [50], random forest [51]. Details on these methods can be found in [52,53].

These geographical distribution techniques are commonly used to estimate the potential distributions of species through a combination of environmental variables and species occurrence [52,53]. Here, we fueled the system with *leaves* occurrence (groups generated by the decision tree) instead of species occurrence (Fig 1), so the cells of grid have information on the presence or absence of the different classes represented by leaves. We opted to use *leaves* in place of landcover categories because the latter show a larger climatic space due to extensive geographic range distribution. The use of *leaves* could avoid over-extrapolation of the spatial extent of landcover because it would minimize the climate range. The climatic variables used were those indicated by the decision tree analysis as being efficient for differentiating and predicting landcover categories. In all likelihood, we increased the robustness of our prediction with this approach by avoiding model overparameterization. The distribution models were analyzed in R software, using the *BIOMOD* package [54].

Our modeling approach started by estimating the best method of prediction under current climatic conditions. We assumed the best method as that for which the predicted distribution (generated through the pre-industrial climatic scenarios) most closely matched the original distribution (i.e. the result of the decision tree classification) (Fig 1). This approach was possible because we knew the original distribution of the focal object of our study (i.e. *leaves* representing the landcover categories), which is not possible for most species distribution studies.

The quality of any spatial modeling approach can be affected by spatial autocorrelation present in both the environmental database, due to gradients of climate variation, and in the biological database, due to a direct response to climate gradients and biases in species sampling [55,56]. To avoid this problem, we modeled the distribution through pre-industrial climatic scenarios using 25% randomly-selected grid cells; the random selection theoretically permitted a sampling free of bias, covering the environmental variability of the study area and improving the model-adjusted predictions [26]. The distributions of *leaves* were generated through 10 replicates of each method, whereby the selected grid cells (the 25% cited above) were divided into 75% of the data for calibration and 25% for validation [26,57,58]. The threshold applied to convert the occurrence probabilities into binary classification was the prevalence of *leaves* in each data calibration, which expresses the prevalence of training data [59]. The ensemble forecasting of distribution modeling methods was generated through averaging of the models’ predictions, weighted by True Skill Statistics (TSS) [60] calculated for internal validation.

Then we compared the predicted distributions and the original distribution of each class using the 75% of grid cells not used for generating the geographic distribution models (Fig 1). This comparison was done through four evaluation metrics: sensitivity, specificity, TSS and Area Under ROC Curve (AUC). The modeling methods with values higher than 0.7 for all evaluation metrics where considered able to estimate the distributions of *leaves*. The distributions for future scenarios were modeled through those methods indicated as adequate according to our criterion and were performed through the same parameters as described above (Fig 1).

After modeling, the *leaves* classified under the same landcover category were aggregated to express the totality of their spatial distribution. Our objective was to infer the effects of climate change on native landcover categories and the comparison would not be possible using the *leaves* because they are not comparable among climatic models.

Finally, a second ensemble forecasting regarding the prediction of the three different GCMs was undertaken to represent the final distribution of each landcover category. We did an ensemble forecasting among GCMs to find the locations where all predictions converged to reduce prediction uncertainties. Then we compared the future landcover distribution with the current distribution to find places where it remained unchanged; these areas can be considered as locations of climatic and landcover stability, which are potential refuges for biodiversity. To do this, the current and future landcover distributions were compared through two descriptors of range, i.e. the area and the location of the latitudinal/longitudinal centroid, to evaluate the changes mediated by climate. We then calculated the potential area of refuges in each South American country. All measures of geographical distributions were calculated using ArcMap® software version 10.

## Results

### Landcover classes and variable selection

According to the random forest results, the decision tree efficiently grouped grid cells (73% of variation was explained in all cases). However, approximately 56% of grid cells were classified differently from their original landcover classes. For these grid cells, predicted landcover was frequently consistent—grid cells from the same original landcover ended in the same class (for example, the *open broadleaved deciduous forest* was frequently predicted as *closed to open shrubs*; S2, S3 and S4 Figs), indicating that these landcover categories overlapped climate boundaries, but still had a climatic identity (S2, S3 and S4 Figs).

**Fig 2:**
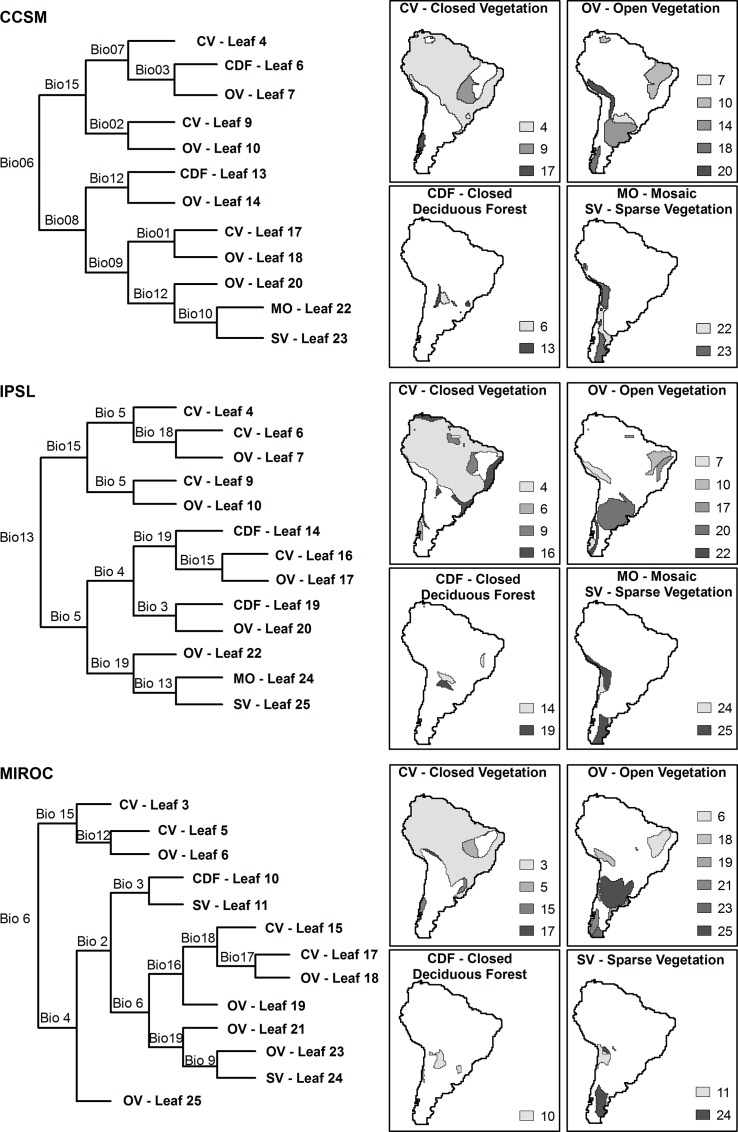
Decision tree and the reclassified landcover. The final *leaves* (subgroups) correspond to the reclassified landcover generated by climatic similarity of native landcover for each climate model used in this study. The final *leaves* can have the same name among different climatic models, but they are not necessarily equivalent. Climatic variable codes are given in S1 Table.

The final aggregation of similar climate-linked landcover types resulted in four categories for MIROC and five for CCSM and IPSL (Fig 2 and S1 Fig). However, most of these categories covered a wide range of climatic conditions and can be divided into subgroups (*leaves*) according to their climatic properties (Fig 2). The grid cells comprising a *leaf* are spatially aggregated, which is expected due to the spatial structure of climatic variation. Thus, the final *leaves* of the decisions tree are subdivisions of spatially aggregated landcover categories (Fig 2).

The *leaves* of the decision tree are different among GCMs because they were structured by different variables and node numbers (Fig 2 and S4 Fig). However, despite variation in decision tree structure, we observed general subdivision trends derived from different climate models (for example CCSM—*Leaf* 4, IPSL–*Leaf* 4 and MIROC–*Leaf* 3; CCSM—*Leaf* 14, IPSL–*Leaf* 20 and MIROC–*Leaf* 25; this kind of matching can be observed for many other *leaves*).

The efficiency of our classification was not affected by anthropogenic landcover. The proportion of grid cells correctly classified was approximately the same when we excluded from the decision tree those cells with more than 50% of area occupied by anthropogenic landcover (0.40–0.45 of grid cells correctly classified). Therefore, the probability of a grid cell to be correctly classified was not related to the proportion of anthropogenic landcover in the cell.

### Landcover distribution and changes mediated by climate

**Fig 3:**
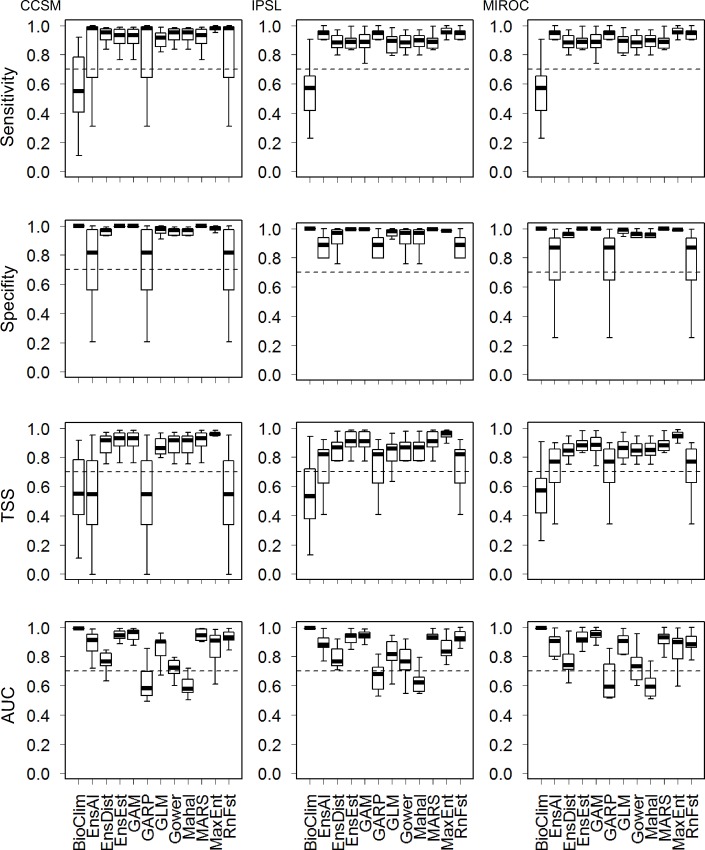
Predicted distributions accuracy and robustness. Evaluation metrics (y-axis) to select the best geographical distribution model approach (x-axis) for predicting current native landcover distributions under three different climatic models (CCSM, IPSL, and MIROC).

We performed 4,560 models of native landcover distribution against pre-industrial climate scenarios (12 modeling methods*10 replicates*38 *leaves*, divided into three climatic models), which generated 456 estimated distributions. However, many of them were inconsistent with original distributions, showing that geographical distribution modeling can fail to estimate geographical distribution (Fig 3). The statistical methods for modeling distributions showed the best performance because they presented values higher than 0.7 for all evaluation metrics, with the exception of GLM (Fig 3). GAM and MARS had similar performance and range distribution predictions. Thus, GAM and MARS strongly influenced the ensemble forecasting of the statistical methods, making the predictions of these three methods largely similar (Fig 3). To simplify comparisons and conclusions, we selected the statistical method of ensemble forecasting to predict landcover distributions under future climate scenarios; in this, it was quite efficient and, being an ensemble, it should generate the best results in terms of predicting distributions.

Unlike species distributions, the predicted landcover distributions should not overlap because only one landcover category can occur in a given location. We considered an uncertainty of the predicted distribution those locations where the occurrence of more than one *leaf* was predicted. The areas of distribution uncertainty were not the same among climatic models (S6 Fig). For predictions generated through the CCSM and MIROC climatic variables, the distribution uncertainty was higher in future climatic scenarios, mainly for RCP 8.5 (S6 Fig). However, the IPSL generated predictions with higher uncertainty for pre-industrial climatic scenarios (S6 Fig). The proportion of uncertainty did not differ widely among *leaves* (S6 Fig), showing that the effectiveness of prediction was not linked to landcover category, which increases the robustness of our approach.

**Fig 4:**
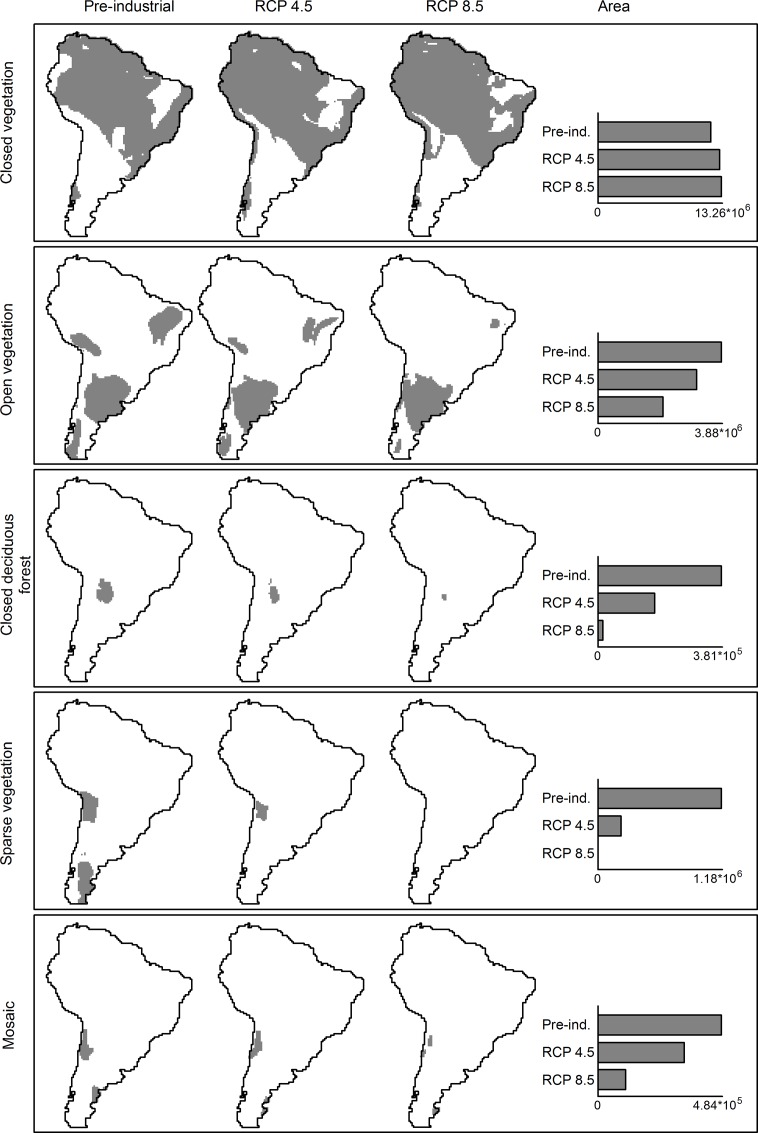
Distribution and losses of native landcover. Spatial distribution and area (km^2^) of native landcover according to the category and climatic scenarios.

**Fig 5:**
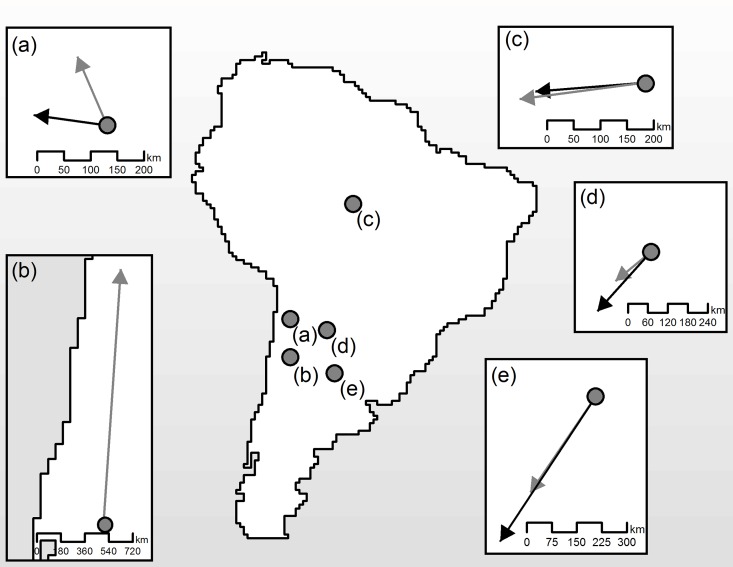
Centroid displacement of landcover mediated by future climate change. In the map of South America we show the location of the centroids for native landcover categories (A—mosaic; B—sparse vegetation, C—closed vegetation, D—open vegetation, E—closed deciduous forest). Insets relate to each native landcover; the points represent the centroids of their respective current distributions, while the arrowheads represent the respective centroids under the 2080 climatic scenarios, showing the direction of change with arrow length representing the intensity of change. The gray arrows refer to RCP 4.5 and black arrows to RCP 8.5.

All landcover categories showed changes in area and displacement of distribution centroids; the changes predicted by RCP 4.5 and RCP 8.5 scenarios were usually in the same direction, but the latter were more intense (Figs 4 and 5). Closed vegetation was the only native landcover predicted to expand. This expansion was mostly in the western part of its distribution, which generated a centroidal displacement in this direction. The other native landcover types tended to severely reduce their range distribution (Fig 4). Open vegetation was predicted to lose range mainly in the Northeast, but with limited expansion in the South, which displaced the centroid south-westwards by more than 300 km. The closed deciduous forest could be reduced by more than 50% in the more optimistic future climatic scenarios (Fig 4), with this range contraction displacing the centroid. Sparse vegetation seemed to be the most affected in terms of range distribution contraction and displacement, shifting almost 2,000 kilometers northwards (Fig 5) or even being totally extirpated under the most pessimistic climate change scenario (Fig 4). Mosaic presented the lowest centroidal displacement (Fig 5), but this is not indicative of reduced concern because the proportion of predicted range contraction was intense, which could reduce the mosaic vegetation to three small and isolated vegetated nuclei (Fig 4).

**Fig 6:**
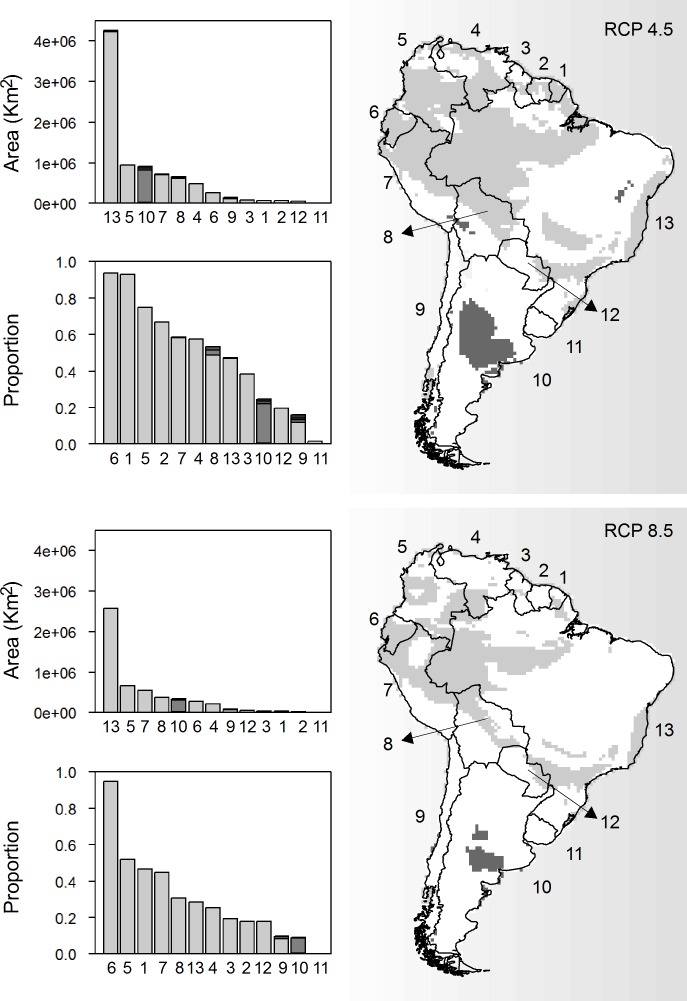
Area, proportion and spatial distribution of climate refuges in 2080 according to two greenhouse gas emissions scenarios (RCP 4.5 and RCP 8.5). Of the five landcover categories analyzed in this study, only three had sufficient area to be graphically represented: closed vegetation (light gray), open vegetation (dark gray), and mosaic (black). South American countries are represented as follows: 1 –French Guyana, 2 –Suriname, 3 –Guyana, 4 –Venezuela, 5 –Colombia, 6 –Ecuador, 7 –Peru, 8 –Bolivia, 9 –Chile, 10 –Argentina, 11 –Uruguay, 12 –Paraguay, and 13 –Brazil.

The ensemble of different climate scenarios showed that 47.3% of South America can be considered areas of climatic stability under the RCP 4.5 scenario. These areas can be considered refuges for biodiversity facing the potential impacts of climate change. However, only 29.1% of the territory was indicated as refuges under the RCP 8.5 scenario. The main landcover types represented in these refuges under both climatic scenarios were closed vegetation (RCP 4.5 = 39.5%; RCP 8.5 = 25.2%), followed by open vegetation (RCP 4.5 = 4.6%; RCP 8.5 = 1.6%; Fig 6). The other landcover categories had limited areas of stability under RCP 4.5 (CDF = 0.001%; SV = 0.4%; MO = 0.3%), which could potentially decrease or even disappear under the RCP 8.5 scenario (MO = 0.1%).

The potential biodiversity refuges are concentrated in the Northwest of the South American territory, occupying a large proportion of Ecuador, Colombia, French Guyana and Peru (Fig 6). However, when we considered the total area, the countries with the largest extent of closed vegetation refuges were Brazil, Colombia, Peru and Bolivia (Fig 6). Argentina accounted for a significant area of open vegetation refuges (Fig 6). Chile and Uruguay presented the most threatened conservation scenarios due to their small area coverage of potential refuges for biodiversity (Fig 6).

## Discussion

Aggregation of native landcover categories by the decision tree resulted in four very broad groups and, consequently, the predictions corresponded more to changes at the biome level than to vegetation classes. This might be a consequence of the Globcover database we used in the model, which does not take topography, an important predictor of vegetation into account [61]. Despite the generality of our results, they reveal some interesting trends.

The distributions of the majority of the native landcover categories grouped by climate are predicted to be reduced in total area, except closed forest. Therefore, there will be losses of total area for four of the five landcover categories climatically defined, which correspond to those that already occupy the smallest areas. Losses of these landcover categories range from approximately 20% under optimistic scenarios (RCP = 4.5) to their complete extirpation under pessimistic scenarios (RCP = 8.5), which could result in the extinction of many species and particularly habitat-specialists. In contrast, area coverage was predicted to increase for closed vegetation. At first, our results seem to contrast with most other studies, which predict that climate change will cause a reduction in forested areas. This outcome is particularly expected in Amazonia, where a savannization process [62–66], through a replacement of forested land by open and/or sparse vegetation is the most supported scenario [62,64,67]. However, this contraction is expected to occur mainly in the central area of South America, where dryer environments are located [68–70]. The expansion of closed forest in our results is predicted mainly in the direction of the West and North coasts of the continent, with some contraction in the middle of the continent where Amazonian savannization is also predicted [71]. However, the savannization hypothesis is still little tested, with supporting evidence only at a local scale [71], demonstrating that more attention should be given to investigating this possibility. By predicting westward expansion of the Amazonian forest, our results bring more uncertainty to this discussion.

There has been a lack of predictions under future climate scenarios for the Brazilian Caatinga biome, located in the Northeast region of South America. This biome is typically composed of open and dry vegetation, sometimes considered as desert [72]. The projection scenarios predict a contraction of open vegetation in that region, bringing uncertainty about the future of open areas in the northeast of the continent. However, here, we have predicted a partial expansion of closed vegetation into Caatinga, which could explain previous evidence of mammal range expansion into the areas presently occupied by the Caatinga, suggesting that mammals could be favored by climate change with concomitant expansion of their present distributions [73]. Therefore, our models seem to indicate that a relaxation of dry environmental conditions in areas of Caatinga could occur, resulting in a landcover that would exhibit an intermediate structure between closed and open vegetation.

Displacement of vegetation types threatens all categories, but is more intense for sparse vegetation, which is also the most affected by range contraction. Predictions for mosaic landcover also estimate a large contraction and displacement in range distribution, considering its total area. Mosaic and sparse vegetation presently are found at the Atacama Desert and other areas of the Andes. Climate change is expected to intensely affect these landcover categories because it will disrupt the extreme weather conditions that regulate the ecological processes and species distributions occurring there. Contraction of the sparse vegetation and mosaics characteristic of the Atacama Desert and Andes could exacerbate the isolation of the specialist species of those habitats, such as the Andean cat (*Leopardus jacobita*), the most threatened felid of the Americas [74,75]. This process of habitat contraction can engender a habitat island effect, with progressive reductions ultimately leading to extirpation, giving no options to species for dispersal, which could drive species extinction and/or alter evolutionary processes and patterns. Thus, sparse vegetation and mosaics, as well as the species restricted to these landcovers, would seem to be the most affected by climate change and, consequently, they are the categories of most concern regarding climatic threats.

Regardless of their direction and intensity, the predicted changes to native landcover distributions are a threat to biodiversity because 2080 (the year for which extrapolations were estimated) is in the near future considering the temporal scales of some ecological and the majority of evolutionary processes. The intensity and velocity of these changes will probably nullify the evolutionary adaptive time of species and their ability to disperse to adequate areas [20]. Despite the evidence of environmental plasticity in some species [20], it is probable that the effect of climatic change on species will be more intense than what we have described here for landcover categories, because they include just very broad categories and we know that there are more variations in vegetation types, mainly at local scales.

Recognizing areas of distributional stability among the different climate models is essential because they are potential refuges for biodiversity. Closed vegetation seems to be the least vulnerable because it is predicted to retain the largest proportion of potential forested refuges [30,76]. However, our results indicate that the spatial distribution of these refuges is concentrated in the Southwest of the Brazilian Amazon and the upper Pacific coast of South America, which includes many distinct vegetation types and biogeographic provinces, found on both sides of the Andean chain. Therefore, with appropriate protection, these forested landcovers will likely maintain climate refuges for biodiversity, while the biodiversity distributed in other areas presently covered by closed forests are threatened, such as the species and ecological processes in the closed vegetation of the Atlantic coast.

While the threats to closed vegetation differ among South America regions, the other landcover categories present a critical conservation situation from the viewpoint of climatic refuges. Open vegetation is the second largest landcover category among those evaluated here, extending from the Northeast through the Mid-South of South America. However, our identified refuges are only a small proportion of its original distribution and these are located predominantly in Argentina. Other landcover types showed even more critical states due to their small distribution so that refuges are almost nonexistent.

The lack of refuges predicted for some landcover categories or regions does not necessarily mean their complete absence. Divergences among climate models may result in failure to identify refuges, particularly for landcover categories with limited distributions. Thus, research at a smaller scale (local, regional or targeted to particular species with restricted ranges) probably will be more sensitive to methodological approaches using climatic variables. Improved climate modeling also would permit better predictions of landcover, ecosystems and species distributions [77], especially those focused at regional scales, such as those of countries. However, improvements to regional model predictions may require better climate data and more careful planning of the distribution of registering stations. For vegetation, a better understanding of climate variability, which at present is little understood, will likely be crucial, because seasonality is critical to define some characteristics of the vegetation types, such as deciduousness [78].

Another possible source of uncertainty, for which we had less control, was the initial database of our modeling. A comparison of different digital databases has shown that Globcover data overestimate the closed forest class [61]. This class occupied the largest part of the original landcover map and the largest predicted area after modeling. We cannot be sure if the initial size of categories could have influenced the estimation of refuges. Unfortunately, despite the uncertainties involved in models and databases, actions for effective conservation cannot await a decreased knowledge gap because the disruption of weather patterns now threatens many species and ecological process [79–81]. Therefore, decisions need to be taken based on the best available information, and, in this sense, areas of refuges predicted here, at least for closed forests, are similar to those predicted in previous work [67,82].

## Supporting Information

S1 Fig Study area and vegetation upscale. (A) Study area and landcover categories according to GlobCover (Bontemps et al., 2011) upscaled according to the most abundant landcover category in grid cells of 0.50 latitude and longitude resolution. (B) The final aggregation of landcovers according to climatic similarity.
(TIF)

S2 Fig Effectivity of reclassification through CCSM. Prediction probability of grid cells from each landcover category based on the CCSM climate model.
(TIF)

S3 Fig Effectivity of reclassification through IPSL. Prediction probability of grid cells from each landcover category based on the IPSL climate model.
(TIF)

S4 Fig Effectivity of reclassification through MIROC. Prediction probability of grid cells from each landcover category based on the MIROC climate model.
(TIF)

S5 Fig Decision tree size. Decision tree sizes were defined by the increase in relative error with inclusion of a new node.
(TIF)

S6 Fig Uncertainty surrounding the potential distribution of leaves as an estimate of prediction error. The bars represent the total area (km^2^) of potential distribution predicted for each leaf (a subdivision of the climatic data generated by the decision tree). In black is the area and location of uncertainty, which relates to a leaf that overlaps the distribution of other leaves.
(TIF)

S1 Table Bioclimatic variables. List of variables used to evaluate climate descriptors and predictors of landcover categories occurring in South America.
(DOCX)
