# Supplementary figures and images for: Impacts of Climate Change on Native Landcover: Seeking Future Climatic Refuges
Source: PLoS One. 2016 Sep 12;11(9):e0162500. doi: 10.1371/journal.pone.0162500 (PMC5019498; doi:10.1371/journal.pone.0162500)

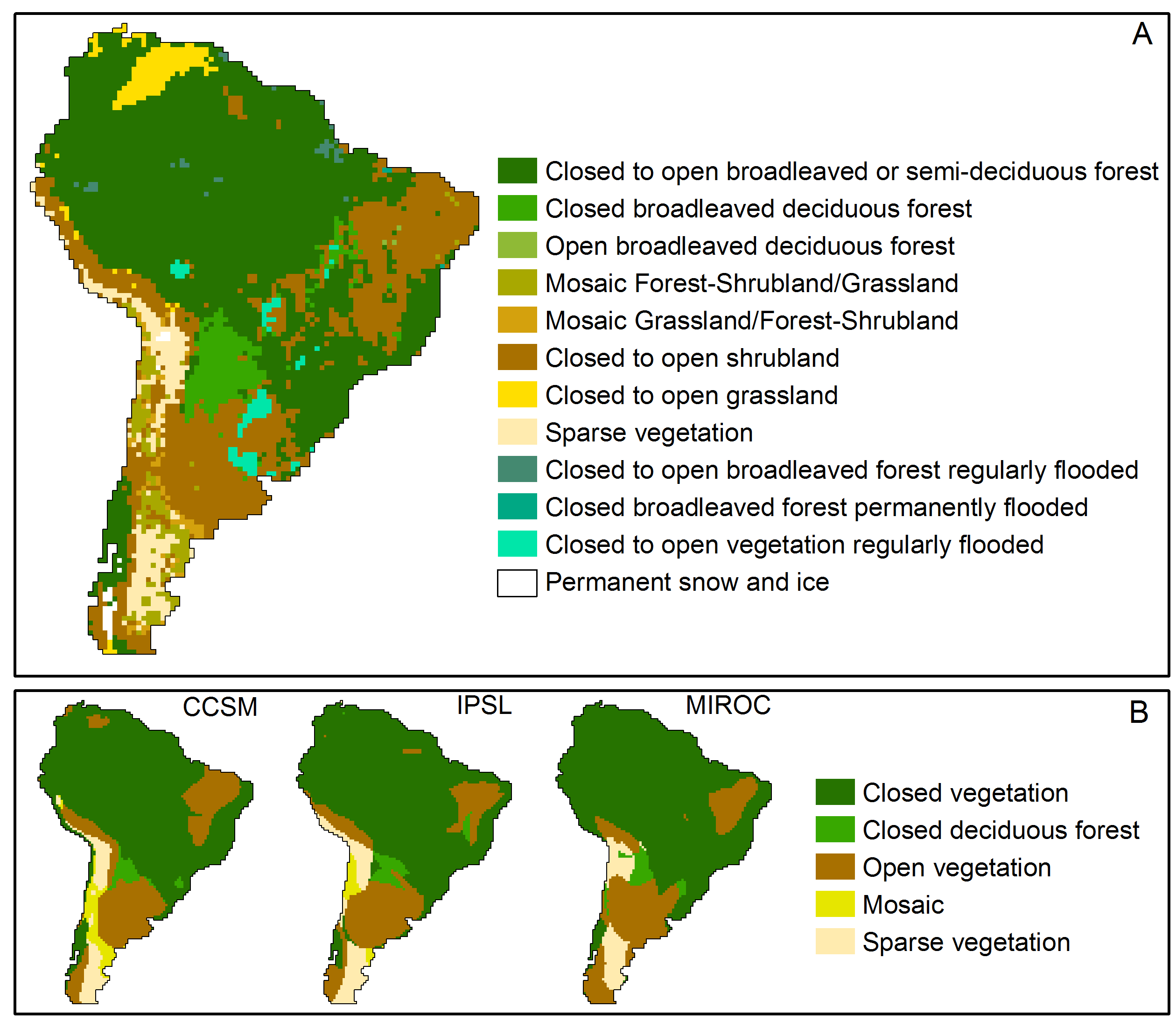

Supplement: S1 Fig — (A) Study area and landcover categories according to GlobCover (Bontemps et al., 2011) upscaled according to the most abundant landcover category in grid cells of 0.50 latitude and longitude resolution. (B) The final aggregation of landcovers according to climatic similarity. (TIF) [file pone.0162500.s001.tif]

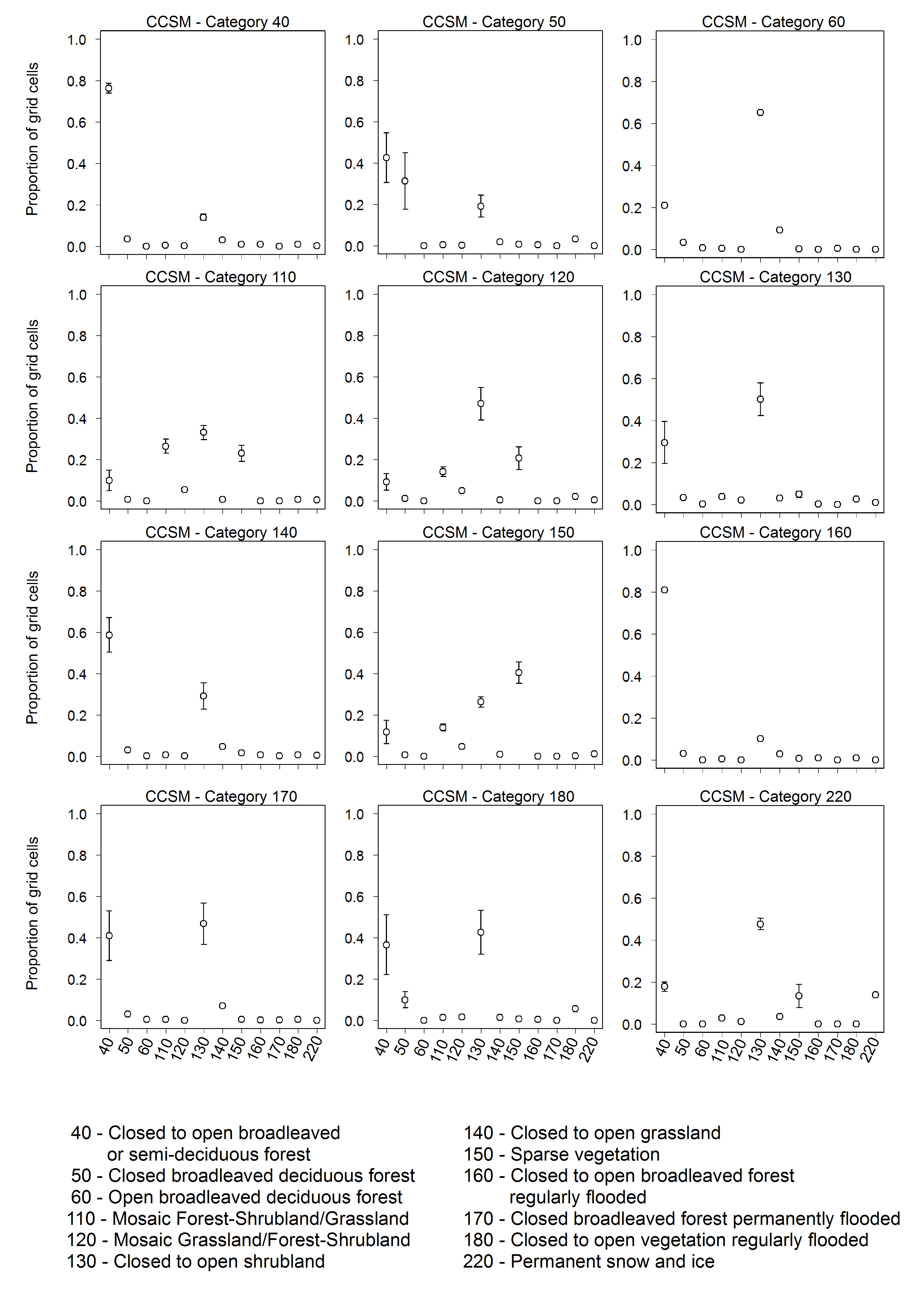

Supplement: S2 Fig — Prediction probability of grid cells from each landcover category based on the CCSM climate model. (TIF) [file pone.0162500.s002.tif]

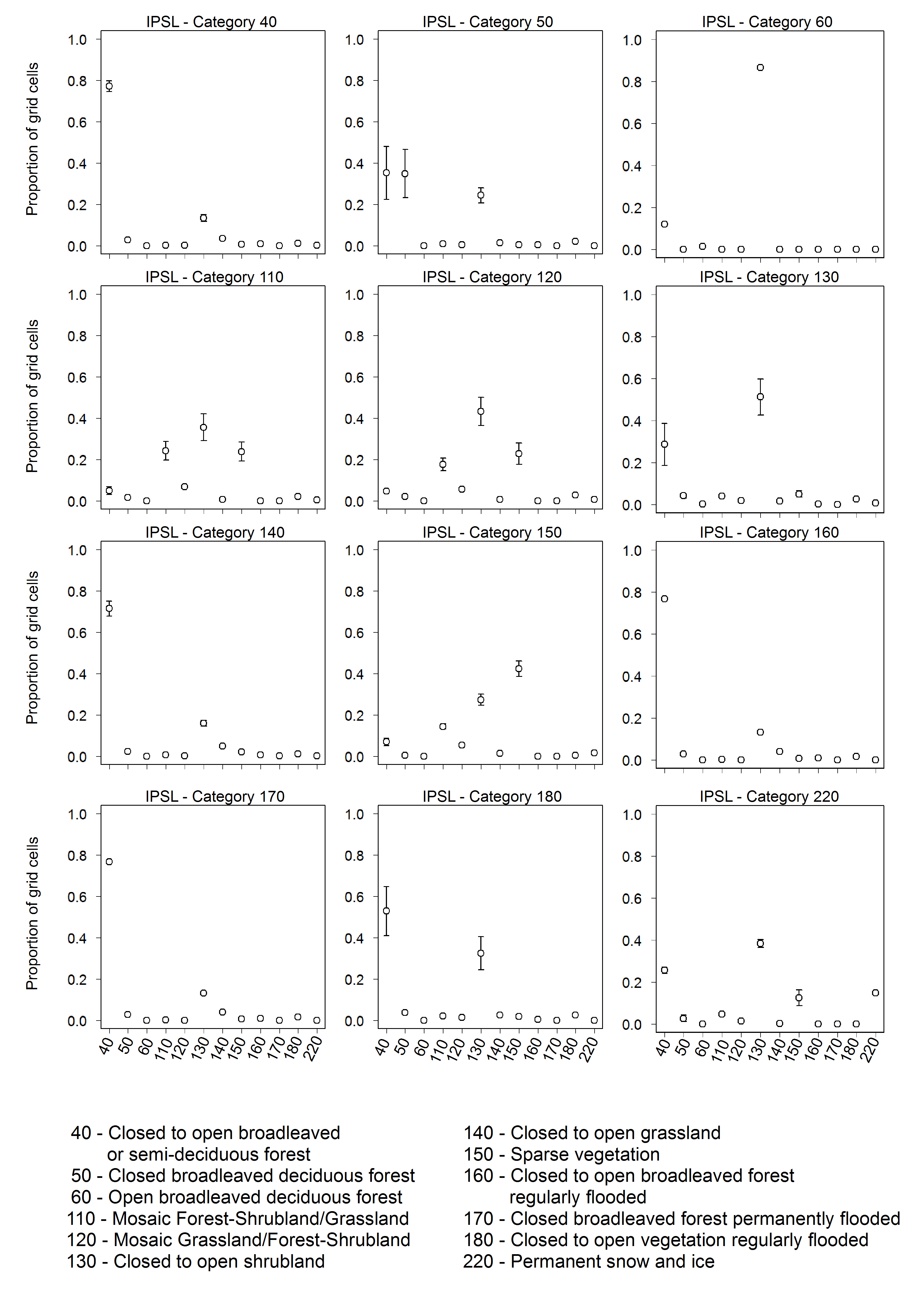

Supplement: S3 Fig — Prediction probability of grid cells from each landcover category based on the IPSL climate model. (TIF) [file pone.0162500.s003.tif]

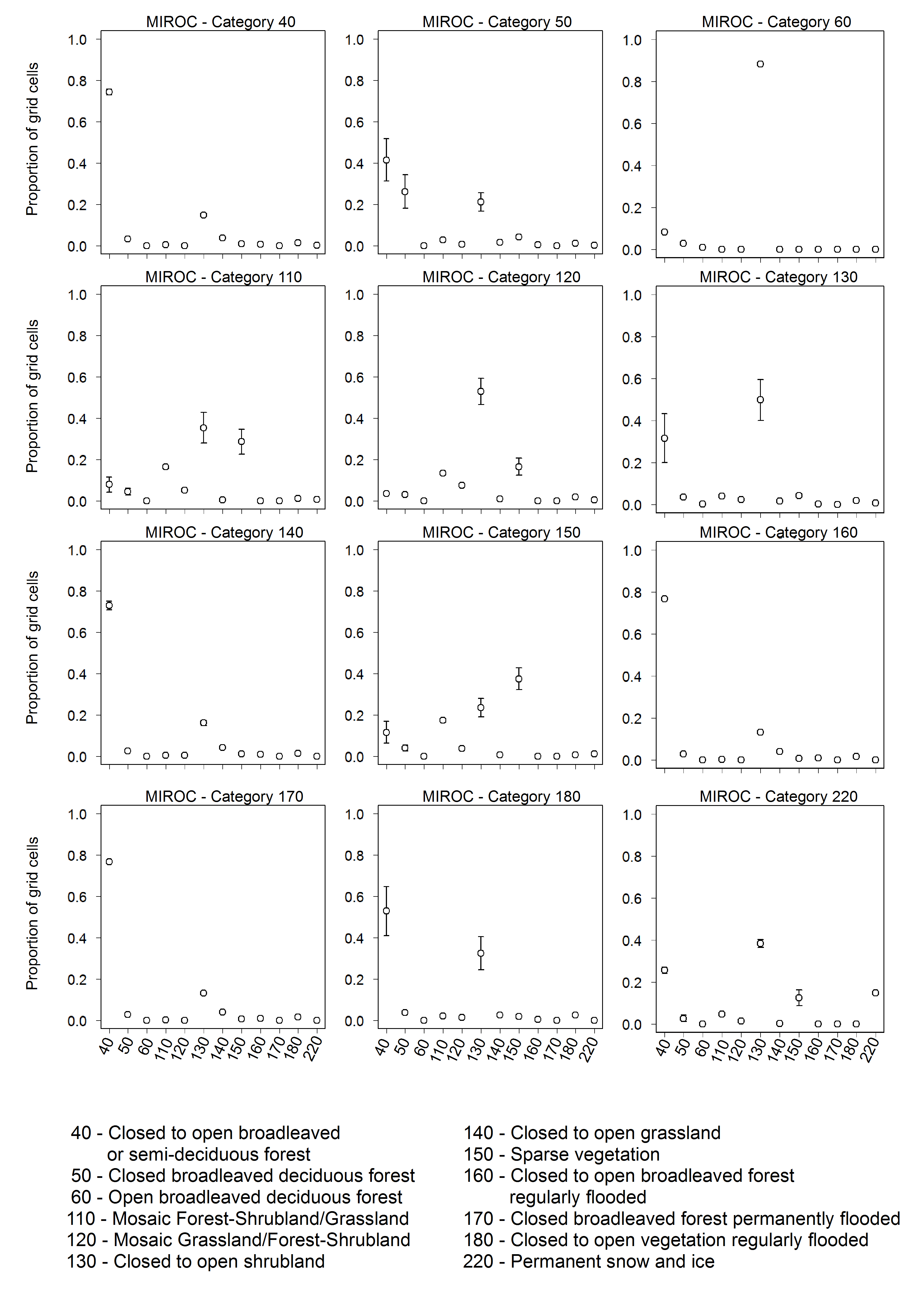

Supplement: S4 Fig — Prediction probability of grid cells from each landcover category based on the MIROC climate model. (TIF) [file pone.0162500.s004.tif]

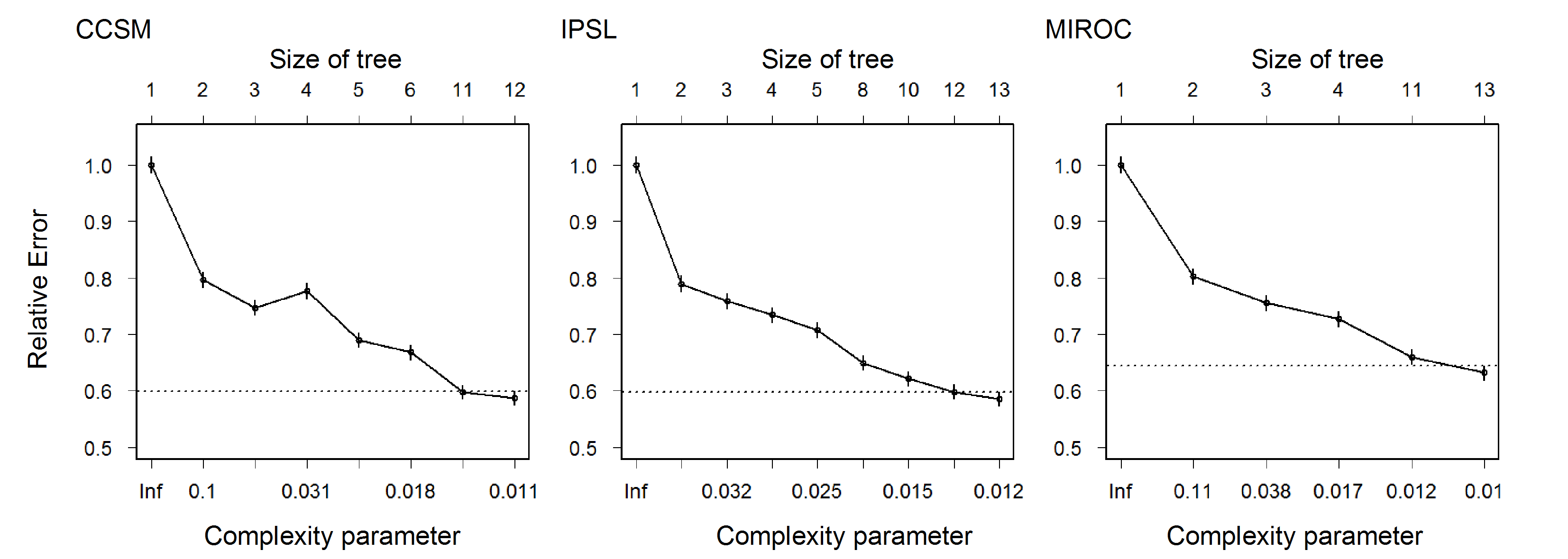

Supplement: S5 Fig — Decision tree sizes were defined by the increase in relative error with inclusion of a new node. (TIF) [file pone.0162500.s005.tif]

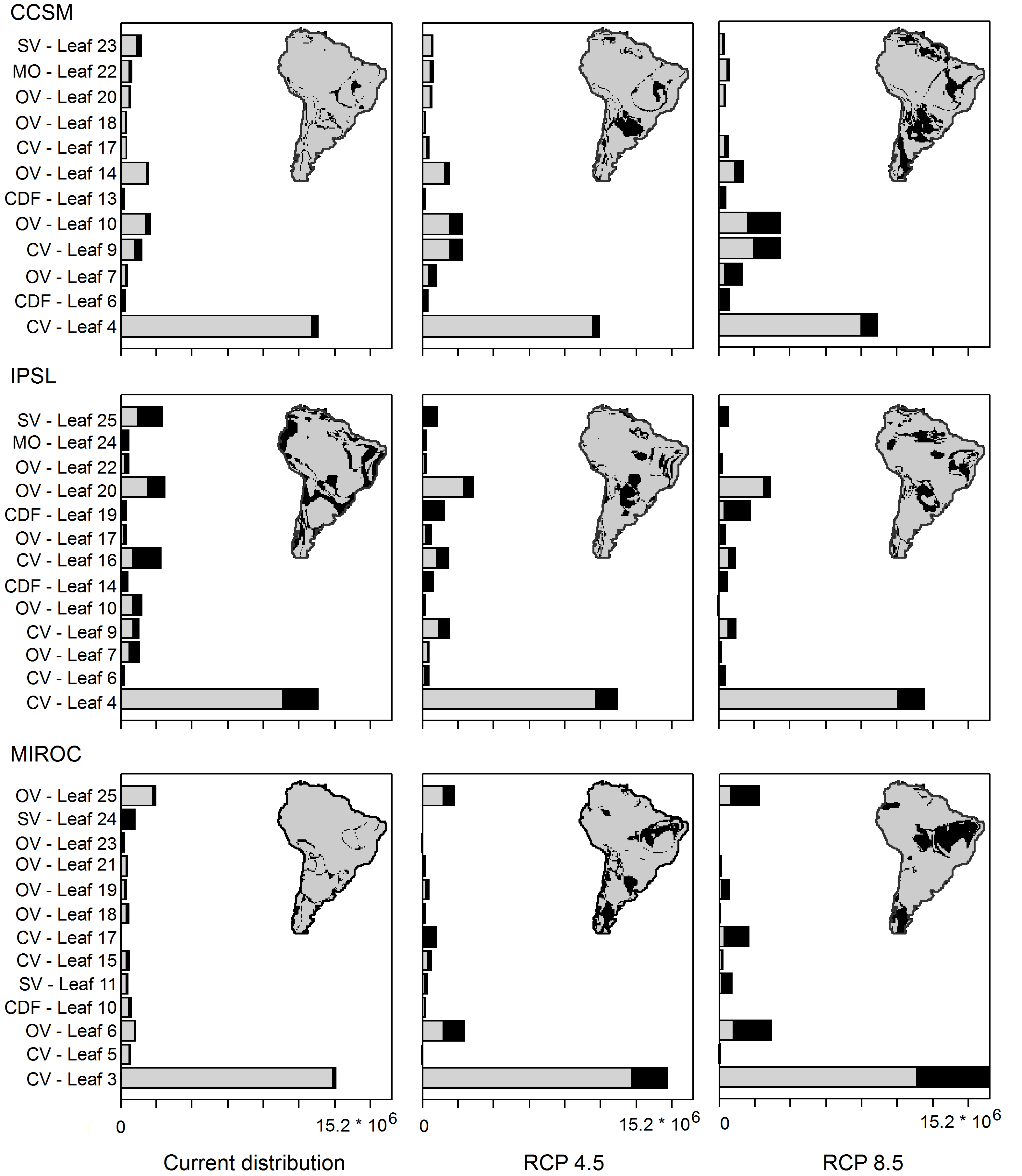

Supplement: S6 Fig — The bars represent the total area (km2) of potential distribution predicted for each leaf (a subdivision of the climatic data generated by the decision tree). In black is the area and location of uncertainty, which relates to a leaf that overlaps the distribution of other leaves. (TIF) [file pone.0162500.s006.tif]
