# Supplementary material for: Impacts of Climate Change on Native Landcover: Seeking Future Climatic Refuges
Source: PLoS One. 2016 Sep 12;11(9):e0162500. doi: 10.1371/journal.pone.0162500 (PMC5019498; doi:10.1371/journal.pone.0162500)
Supplement: S1 Table — List of variables used to evaluate climate descriptors and predictors of landcover categories occurring in South America. (DOCX) [file pone.0162500.s007.docx]

S1 Table – List of variables used to evaluate climate niches of landcover categories occurring in South America.

| Code | Description |
| --- | --- |
| Bio1 | Annual mean temperature |
| Bio2 | Mean diurnal range (mean of monthly (max temp - min temp)) |
| Bio3 | Isothermality (Bio2/Bio7) (* 100) |
| Bio4 | Temperature seasonality (standard deviation *100) |
| Bio5 | Max temperature of warmest month |
| Bio6 | Min temperature of coldest month |
| Bio7 | Temperature annual range (Bio5-Bio6) |
| Bio8 | Mean temperature of wettest quarter |
| Bio9 | Mean temperature of driest quarter |
| Bio10 | Mean temperature of warmest quarter |
| Bio11 | Mean temperature of coldest quarter |
| Bio12 | Annual precipitation |
| Bio13 | Precipitation of wettest month |
| Bio14 | Precipitation of driest month |
| Bio15 | Precipitation seasonality (coefficient of variation) |
| Bio16 | Precipitation of wettest quarter |
| Bio17 | Precipitation of driest quarter |
| Bio18 | Precipitation of warmest quarter |
| Bio19 | Precipitation of coldest quarter |
